#  “Budget Impact Analyses”: A Practical Policy Making Tool for Drug Reimbursement Decisions

**Published:** 2014

**Authors:** Hamid Reza Jamshidi, Naghmeh Foroutan, Jamshid Salamzadeh

**Affiliations:** a *Department of Pharmacoeconomics and Pharmaceutical Management, School of Pharmacy, Shahid Beheshti University of Medical Sciences, Tehran, Iran.*

## Abstract

In the present article, Budget Impact Analysis as an effective, practical financial tool has been introduced to the policy makers for improving drug formulary and reimbursement decision making. In Iran, Ministry of Health (MOH), health insurance organizations, and health care providers such as hospitals could take the most advantage of the BIAs reports.

## Introduction

A budget impact analysis (BIA) estimates fiscal consequences of adopting a new health technology or intervention within a specific health context (1; 2; 3). Nowadays, in almost every developed country, regulatory and reimbursement authorities increasingly require Budget Impact Analyses (BIAs), along with a Cost-effectiveness analysis (CEA), as part of a formulary listing or reimbursement submission. A BIA can also be useful in budget or resource planning process. A BIA as a part of a comprehensive economic assessment has been increasingly used in strategic budget planning in almost every developed country. 

## Experimental

*What is a budget impact analysis?*

A budget impact analysis (BIA) estimates financial consequences of adopting a new health technology or intervention within a specific health context (1; 2; 3). According to the ISPOR task force report II (2014), a standard budget impact analysis model should contain features which have been summarized in Table 1. 

**Table 1:** A Standard Budget Impact Analysis Model Features

Budget Impact Model Features (2)
**Features of the health care system** **Perspective** **Use and cost of current and new interventions** ** Target population** ** Current alternative interventions** ** New intervention and market effects** ** Off-label uses of the new intervention** ** Cost of the current or new intervention mix** **Impact on other costs** ** Condition-related costs** ** Indirect costs** **Time horizon** **Time dependencies and discounting** **Choice of computing framework** **Uncertainty and scenario analysis** **Validation**

International standard guidelines and empirical studies on BIAs have been conducted over the last decade and nowadays many developed countries have included a request for BIA alongside the CEA from pharmaceutical companies when submitting evidence to support national or local formulary approval or reimbursement (4). Some countries have developed their own guidelines and others are doing the analyses in accordance with ISPOR (International Society for Pharmacoeconomics and Outcomes Research) standard guideline (1; 2). Mauskopf *et al. *published an analytic framework for the first time as budget impact modeling in 1998 (5). Since the 1990s, several regions in the world including Australia, North America (Canada, United States) and Europe (England and Wales, Belgium, France, Hungary, Italy, Poland) have included a request for BIA alongside the CEA when submitting evidence to support national or local formulary approval or reimbursement (4). The increasing demand from the payers for evidence of BIAs in parallel to CEA in different countries has motivated the publication of a standard guideline for good practice in BIAs by International Society for Pharmacoeconomics and Outcomes Research (ISPOR) (1), which has provided a backbone for doing a standard BIA worldwide. Although ISPOR guideline is considered a standard template for conducting, reporting and analyzing BIAs, it only provides a general approach for the analyses; thus, each country is required to adapt the model on the basis of its current local financing structure, process, rules and regulations. Canadian (6) and Polish (7) standard guidelines are the best examples in this context. The most important published guidelines have been summarized in Table 2. 

**Table 2:** The most important published international BIA guidelines (2001- 2014).

**Title **	**Country **	**Authors **	**Year **	**Journal **
Principles of Good Practice for Budget Impact Analysis: Report of the ISPOR Task Force on Good Research Practices—Budget Impact Analysis (2)	ISPOR task force	Mauskopf, *et al. *	2014	Value in Health
Developing Guidance for Budget Impact Analysis (3)	UK	Trueman, Drummond and Hutton	2001	Pharmacoeconomics
Guidelines for Conducting Pharmaceutical Budget Impact Analyses for Submission to Public Drug Plans in Canada (6)	Canada	Marshall, *et al. *	2008	Pharmacoeconomics
Proposal of Polish Guidelines for Conducting Financial Analysis and Their Comparison to Existing Guidance on Budget Impact in Other Countries (7)	Poland	Orlewska and Mierzejewski	2004	Value in Health

Regarding empirical studies, numerous pharmaceutical BIA studies were published, mainly from the USA, France, Spain, Ireland, Italy, Denmark, Finland, Thailand, Japan and Belgium (8-16). The analyses covered quite wide variations in terms of diseases (*e.g. *rheumatoid Arthritis, breast cancer, atopic dermatitis, agonist Opioid treatment, Asthma, chemotherapy-induced anemia, Glaucoma, heart Failure, *etc*.). Very few developing countries, especially from the Middle East region, has published and probably developed such analyses; thus, the importance and potential practical benefits of BIA studies in improving efficiency of financial resource allocation in the health sector, especially in developing, low and middle income countries, should be highlighted. 

In Iran, the first pharmaceutical budget impact analysis has been published by Foroutan and colleagues in 2013 on evaluating the budgetary impact of using mTOR-inhibitors (Sirolimus) as immunosuppressive medications in replace to Calcineurin Inhibitors (Cyclosporine) in renal transplantation therapy for the health insurance organizations (17). For doing such analysis in accordance with ISPOR standard guideline, at very first step, cost of renal transplantation therapy (current cost of illness) in Iran has been calculated using cyclosporine as the main immunosuppressive medication (18). Further studies would be required to localize this standard international model in accordance with Iranian health care financing system and policy makers` opinions. 

## Results and Discussion

*A practical policy making implication of BIAs in Iran*

Increasing accessibility and affordability of healthcare services have been considered as important policy objectives since the beginning of 1980s in Iran. However, current almost 70% health care out-of-pocket payments create a barrier to an equal access to quality health services, especially in terms of new medicines which affect equity issues and “health” in Iran (19).

In the recent years, because of economic crises, health care policy makers have faced much more difficulties in allocating limited available budget to several diseases. Currently, cost of medical expenditure is rapidly growing and becoming increasingly unaffordable, even for the payers; and consequently, out-of-pocket (OOP) payments are dramatically growing over time. Health care catastrophic expenses have make health services quite unaffordable for many patients with health threatening diseases (19, 20). 

**Figure 1:**
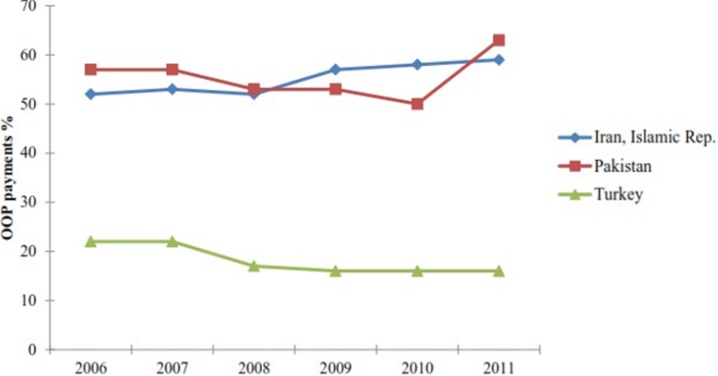
Health care out-of-pocket expenditure in three countries; Iran, Pakistan, Turkey (21).

Figure 1 compares percentage of health care OOP expenditure in three countries of the region; Iran, Turkey and Pakistan, over the last 6 years (21). From the figure, it is clear that Turkey with total health expenditure of almost $52 billion (about $700 per capita and 16% OOP expenditure) had almost 27% of Iran᾽s OOP payments in 2011 while Pakistan had almost the same amount of OOP payments with only $6 billion health care expenditure (Table 3, Figure 1). In case of Iran and Pakistan, the growing trend of health care OOP expenditure should be recognized (Figure 1).

**Figure 2:**
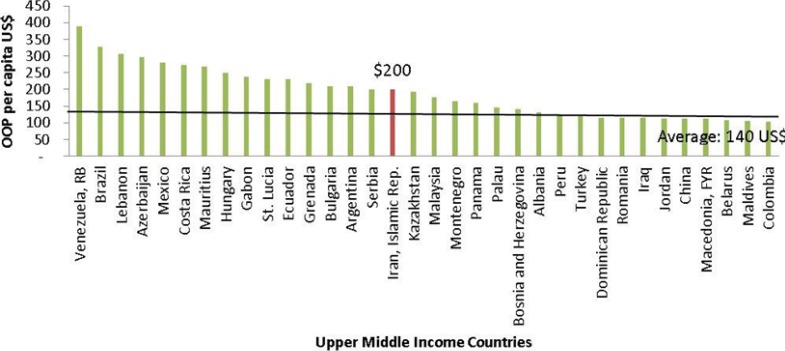
OOP payments in upper middle income countries (2011) (21).

On the other hand, according to the World Bank database, Iran has taken the 1^st^ position in the Middle East region and 16^th^ among upper middle income countries regarding the amount of annual OOP payments per capita in US Dollars (Figure 2).

**Table 3:** Growth indicators in three countries; Iran, Pakistan, Turkey (latest available data; 2011- 2012) (21).

**Indicators**	**Iran**	**Turkey**	**Pakistan**
Land area (sq. km)	1,628,550	769,630	770,880
Population, total	74,798,599	73,639,596	176,745,364
GDP (billion current US$)	514	770	210
GDP per capita (current US$)	6,815	10,666	1,290
GDP growth (annual %)	2	2.2	4.2
Population growth (annual %)	1.3	1.3	1.7
Out-of-pocket health expenditure (% of total expenditure on health)	59	16	63
Out-of-pocket health expenditure, total (billion current US$)	18	8	4
Out-of-pocket health expenditure per capita (current US$)	240	113	30
Out-of-pocket health expenditure (% of private expenditure on health)	97	64	86
Health expenditure per capita (current US$)	346	696	30
Health expenditure, private (% of GDP)	4	2	2
Health expenditure, public (% of GDP)	2	5	1
Health expenditure, public (% of government expenditure)	10	13	4
Health expenditure, public (% of total health expenditure)	40	75	27
Health expenditure, total (% of GDP)	6	7	3
Health expenditure, total (billion current US$)	30	52	6
Life expectancy at birth, total (years)	73	74	65

## Conclusion

Considering the fact that conducting Budget Impact analyses would improve health care resource allocation and would accelerate and facilitate the process of evidence-based reimbursement decision making. We sincerely believe that this method could effectively address the problem of considerable out-of-pocket payments in Iran and would make a great change in drug affordability for different payers.
